# Body composition and risk for metabolic alterations in female adolescents

**DOI:** 10.1590/0103-0582201432215313

**Published:** 2014-06

**Authors:** Eliane Rodrigues de Faria, Cristiana Araújo Gontijo, Sylvia do Carmo C. Franceschini, Maria do Carmo G. Peluzio, Silvia Eloiza Priore

**Affiliations:** 1 Universidade Federal do Espírito Santo (UFES), Alegre, ES, Brasil; 2 UFV, Viçosa, MG, Brasil

**Keywords:** body composition, adolescent, metabolic syndrome x, insulin resistance

## Abstract

**OBJECTIVE:**

To study anthropometrical and body composition variables as predictors of risk for metabolic alterations and metabolic syndrome in female adolescents.

**METHODS:**

Biochemical, clinical and corporal composition data of 100 adolescents from 14 to 17 years old, who attended public schools in Viçosa, Southeastern Brazil, were collected.

**RESULTS:**

Regarding nutritional status, 83, 11 and 6% showed eutrophia, overweight/obesity and low weight, respectively, and 61% presented high body fat percent. Total cholesterol presented the highest percentage of inadequacy (57%), followed by high-density lipoprotein (HDL - 50%), low-density lipoprotein (LDL - 47%) and triacylglycerol (22%). Inadequacy was observed in 11, 9, 3 and 4% in relation to insulin resistance, fasting insulin, blood pressure and glycemia, respectively. The highest values of the fasting insulin and the *Homeostasis Model Assessment-Insulin Resistance *(HOMA-IR) were verified at the highest quartiles of body mass index (BMI), waist perimeter, waist-to-height ratio and body fat percent. Body mass index, waist perimeter, and waist-to-height ratio were the better predictors for high levels of HOMA-IR, blood glucose and fasting insulin. Waist-to-hip ratio was associated to arterial hypertension diagnosis. All body composition variables were effective in metabolic syndrome diagnosis.

**CONCLUSIONS:**

Waist perimeter, BMI and waist-to-height ratio showed to be good predictors for metabolic alterations in female adolescents and then should be used together for the nutritional assessment in this age range.

## Abstract (es)

**OBJETIVO:**

Estudiar variables antropométricas y de composición corporal como pronósticos de riesgo de alteraciones metabólicas y de síndrome metabólico en adolescentes del sexo femenino.

**MÉTODOS:**

Se recogieron datos bioquímicos, clínicos y de composición corporal de 100 adolescentes de 14 a 17 años de escuelas públicas de Viçosa, Minas Gerais (Brasil).

**RESULTADOS:**

Respecto al estado nutricional, 83, 11 y 6% presentaron eutrofia, sobrepeso/obesidad y bajo peso, respectivamente, y 61% presentaron alto porcentaje de grasa corporal. El colesterol total fue el que presentó mayor porcentaje de inadecuación (57%), seguido del HDL (*high-density lipoprotein* - 50%), del LDL (*low-density lipoprotein* - 47%) y de los triglicéridos (22%). Se observó inadecuación en 11, 9, 3 y 4% respecto a la resistencia a la insulina, insulina en ayuno, presión arterial y glucemia, respectivamente. Se encontraron mayores valores de insulina de ayuno y HOMA-IR (índice de resistencia a la insulina, del inglés *Homeostasis Model Assessment-Insulin Resistence*) en los mayores cuartiles de índice de masa corporal (IMC), perímetro de la cintura, relación cintura/estatura y porcentaje de grasa corporal. Se observó que el IMC, el perímetro de la cintura y la relación cintura/estatura fueron las variables que demostraron predecir mejor el diagnóstico de niveles elevados de HOMA-IR, glucemia e insulina de ayuno y la relación cintura/cuadril para diagnóstico de la hipertensión arterial. Todas las variables de composición corporal fueron eficaces en el diagnóstico del síndrome metabólico.

**CONCLUSIONES:**

Las variables perímetro de la cintura, IMC y relación cintura/estatura fueron buenos pronósticos de alteraciones metabólicas en las adolescentes y, por ello, se debe usarlas en conjunto para evaluar el estado nutricional en esa franja de edad.

## Introduction

The World Health Organization defines adolescence as the period between ten and 19 years of age. During this period, individuals undergo physical, psychic, and social changes, which may manifest at different times and in different ways for each person^(^ 1 ^)^. Adolescence is one of the critical periods for the onset or persistence of obesity, as well as for the development of associated complications.

Adolescent and childhood obesity have been reaching epidemic proportions worldwide. Data from the Brazilian Household Budget Survey (Pesquisa de Orçamentos Familiares, POF), conducted between 2008 and 2009, showed that 20.5% of Brazilian adolescents between ten and 19 years old are overweight^(^ 2 ^)^. 

Use of anthropometric measurements to assess cardiovascular risk in adolescents has been little investigated. Among body composition variables, body fat percentage, waist and hip circumferences, and waist-to-hip ratio are the most widely used in studies on this age group^(^ 3 ^-^ 6 ^)^.

Considering that early onset of cardiovascular risk factors increases the risk of cardiovascular diseases in adulthood, identifying simple and noninvasive anthropometric measurements associated with these factors in healthy adolescents is very useful in the prevention of chronic noncommunicable diseases^(^ 3 ^)^. Excess weight and/or body fat may increase the risk of metabolic alterations such as dyslipidemias, insulin resistance, impaired glucose tolerance, and hypertension. When concurrent, these factors support the diagnosis of metabolic syndrome^(^ 4 ^)^.

This study aimed to analyze body composition variables as predictors of the risk of metabolic alterations and metabolic syndrome in female adolescents. 

## Method

This epidemiological cross-sectional study included 100 female students between 14 and 17 years old selected from public high schools in Viçosa, MG, Brazil. Data were collected between September 2006 and January 2007. Inclusion criteria were: having had menarche at least one year prior to the study; having no chronic diseases; taking no medicines that could affect blood pressure, fasting glycaemia or lipid metabolism; not taking contraceptives for less than two months; not taking diuretics/laxatives regularly; and not having a pacemaker and/or a prosthesis. 

Sample selection was based on the total number of female adolescents belonging to the desired age group in 2006 and studying in schools in the metropolitan area of Viçosa, Brazil^(^ 7 ^)^. The sample was calculated using Epi Info 6.04d for cross-sectional studies. We considered a total population of 2,500, an expected metabolic syndrome rate of 8%, and a variability of 2.5%. The sample consisted of 90 people and a 95% level of confidence, and was subsequently increased in 10% to compensate for possible withdrawals from the study. Of all adolescents who fitted the inclusion criteria (n=336), 100 were randomly selected. 

The subjects were weighted on a digital electronic scale with maximum capacity of 136kg, 100g. The adolescents were weighted on a digital scale with a maximum capacity of 136kg and sensitivity of 100g. Height was measured using a 2m stadiometer with 0.1cm resolution, plastic screen, and sliding head piece attached to one extremity. Height and weight were recorded according to techniques recommended by the World Health Organization^(^ 8 ^)^. Nutritional status was evaluated using the BMI, with cutoff points and anthropometric references as defined by the World Health Organization^(^ 9 ^)^. Overweight and obese subjects were classified as overweight (≥ 85 percentile)^(^ 4 ^,^ 10 ^)^. Waist (WC) and hip circumferences (HC) were measured with a 2m flexible nonelastic measuring tape with graduations in cm and mm. During the procedure, the subjects' soft parts were not compressed. The waist-to-hip ratio (WHR) and waist-to-height ratio (WHtR) were calculated. Body fat percentage (BF%) was assessed using a horizontal tetrapolar biompedance measuring device (Biodynamics^(r)^, model 310, version 7.1) and considering Lohman's cutoff points^(^ 11 ^)^. The adolescents were evaluated between 7 a.m. and 8:30 a.m., always following the procedures required prior to the test^(^ 12 ^)^. We applied the validated equation proposed by Houtkooper et al^(13)^ for adolescents between ten and 19 years old. The resistance obtained by biompedance was measured in ohms (Ω).

Blood pressure was measured with an automatic inflation blood pressure monitor, as recommended by the Brazilian Society of Cardiology and as described in the V Brazilian Guidelines for Arterial Hypertension^(^ 14 ^)^. Regarding cutoff points for systolic and diastolic blood pressure, we considered the values based on height percentile. Blood was collected after a 12-hour fasting for assessing glycemia and plasma insulin levels. Serum lipid concentrations, such as total cholesterol, triglycerides, HDL, LDL, and very low-density lipoprotein, were also evaluated. In cases of dyslipidemia and altered insulin resistance (≥15µU/mL), cutoff points for adolescents were in accordance with the I Guideline for Preventing Atherosclerosis in Childhood and Adolescence^(^ 15 ^)^. For altered fasting glycemia, we followed the recommendations from the American Diabetes Association^(^ 16 ^)^, which uses as criterion a fasting glycemia ≥100mg/dL. Resistance to insulin was determined by the Homeostasis Model Assessment-Insulin Resistance (HOMA-IR) method - [fasting insulin (µU/mL) x fasting glycemia (mmol/L)/22.5]≥3.16^(^ 17 ^)^.

We followed the criteria proposed by the International Diabetes Federation (IDF) for the classification of metabolic syndrome while using the cutoff points defined by the Brazilian Society of Cardiology for adolescents: waist circumference ≥90th percentile plus two alterations: triglycerides ≥100mg/dL; HDL <45mg/dL; altered fasting glycemia ≥100mg/dL; blood pressure ≥90th percentile for height and sex.

This study was approved by the Human Research Ethics Committee of the Universidade Federal de Viçosa (ref. no. 013/2006). Participants voluntarily agreed to take part in the study after being informed verbally and through a written consent form. Authorization was obtained from both adolescents and their parents and/or guardians.

Data were analyzed using the Epi Info software, version 6.04d, and the Sigma Statistic^(r)^ for Windows. The following tests were used: Pearson's or Spearman's correlation, Analysis of Variance (ANOVA), or the Kruskal-Wallis test followed by the Dunn or Tukey test, depending on the characteristics of the variables. Significance level was set at 5% (*p*<0.05). The ROC (receiver operating characteristic) curves were established with the Medcalc 9.03 software in order to evaluate the body composition variables that can predict biochemical, blood pressure, and metabolic syndrome alterations. The areas under the curve (AUC) and their confidence intervals were calculated. 

## Results

The mean age of the subjects was 16.0±0.7 years. The median age was 16 years old. Mean age of menarche was 12.3±1.1 years, and median age was 12.1 years (minimum 10, maximum 15.1). Regarding nutritional status, 83% of the adolescents had normal weight, 11% were overweight or obese, and 6% were underweight. Body fat percentage was elevated in 61% of them. 

Total cholesterol levels were inadequate in most subjects (57%), followed by HDL (50%), LDL (47%), and triglicerides (22%). Fasting insulin was altered in 9% of adolescents, and fasting glycemia, in 4% of them. The HOMA-IR was elevated in 11% of subjects, and blood pressure was inadequate in 3%. Metabolic syndrome was present in 3% of the adolescents.

Comparison between mean (standard deviation, SD) and median values of metabolic characteristics was performed according to BMI, WC, WHtR, BF%, and WHR quartiles. Table 1 shows only the biochemical variables that were different between quartiles. The highest values of fasting insulin and HOMA-IR were observed in the upper quartiles of BMI, WC, WHtR, and BF%, and the highest values of total cholesterol/HDL and LDL/HDL were present in the upper quartile of BF%.

**Table 1:**
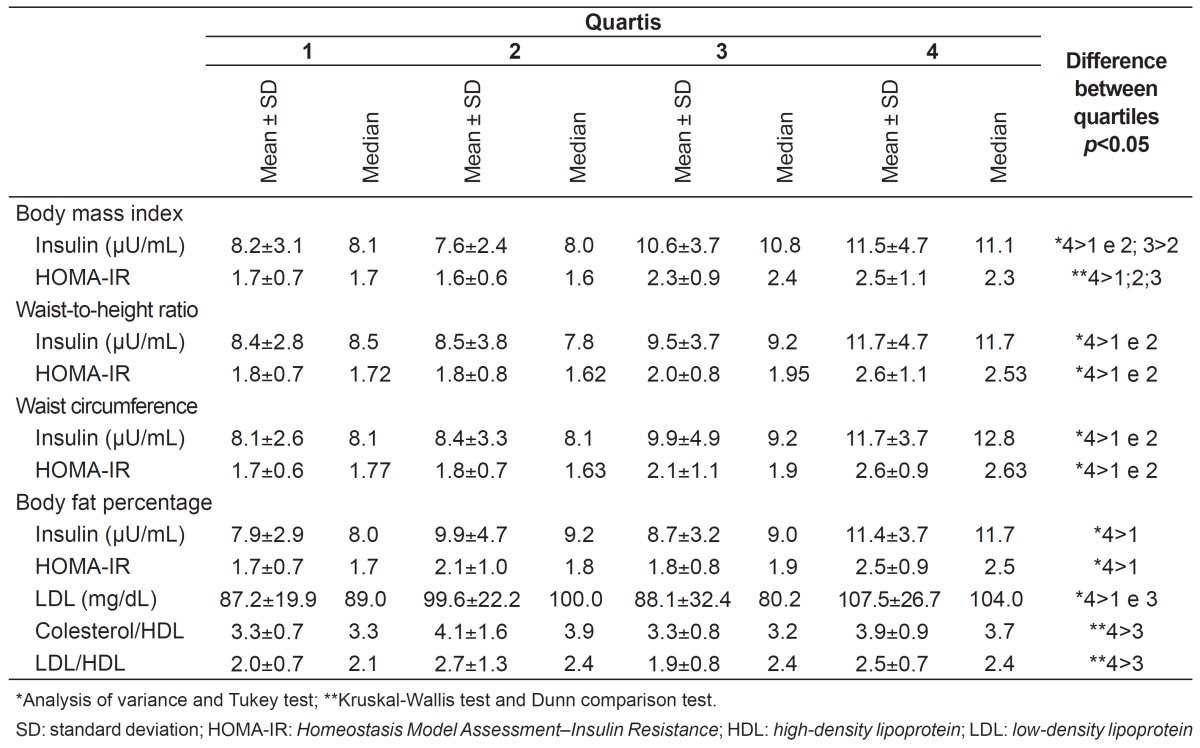
Comparison between mean and median values of metabolic characteristics of adolescents classified into quartiles of body composition variables

A correlation was detected (*p*<0.05) between WHtR and HOMA-IR (r=0.337), insulin (r=0.327), total cholesterol (r=0.256), LDL (r=0.264), total cholesterol/HDL (r=0.265), LDL/HDL (r=0.281), and systolic pressure (r=0.200); between WC and HOMA-IR (r=0.332), insulin (r=0.312), systolic pressure (r=0.238), and diastolic pressure (r=0.252); between BMI and HOMA-IR (r=0.370), insulin (r=0.371), and systolic pressure (r=0.226); between BF% and HOMA-IR (r=0.286), insulin (r=0.303), and total cholesterol (r=0.197); and between WHR, total cholesterol/HDL (r=0.238), and LDL/HDL (r=0.236).

The ROC curves (Table 2) showed that the anthropometric and body composition variables had a smaller AUC for the diagnosis of altered levels of HDL and total cholesterol. Values next to 0.5 showed that these variables are not efficient for the diagnosis of alterations in lipid concentrations. 

**Table 2:**
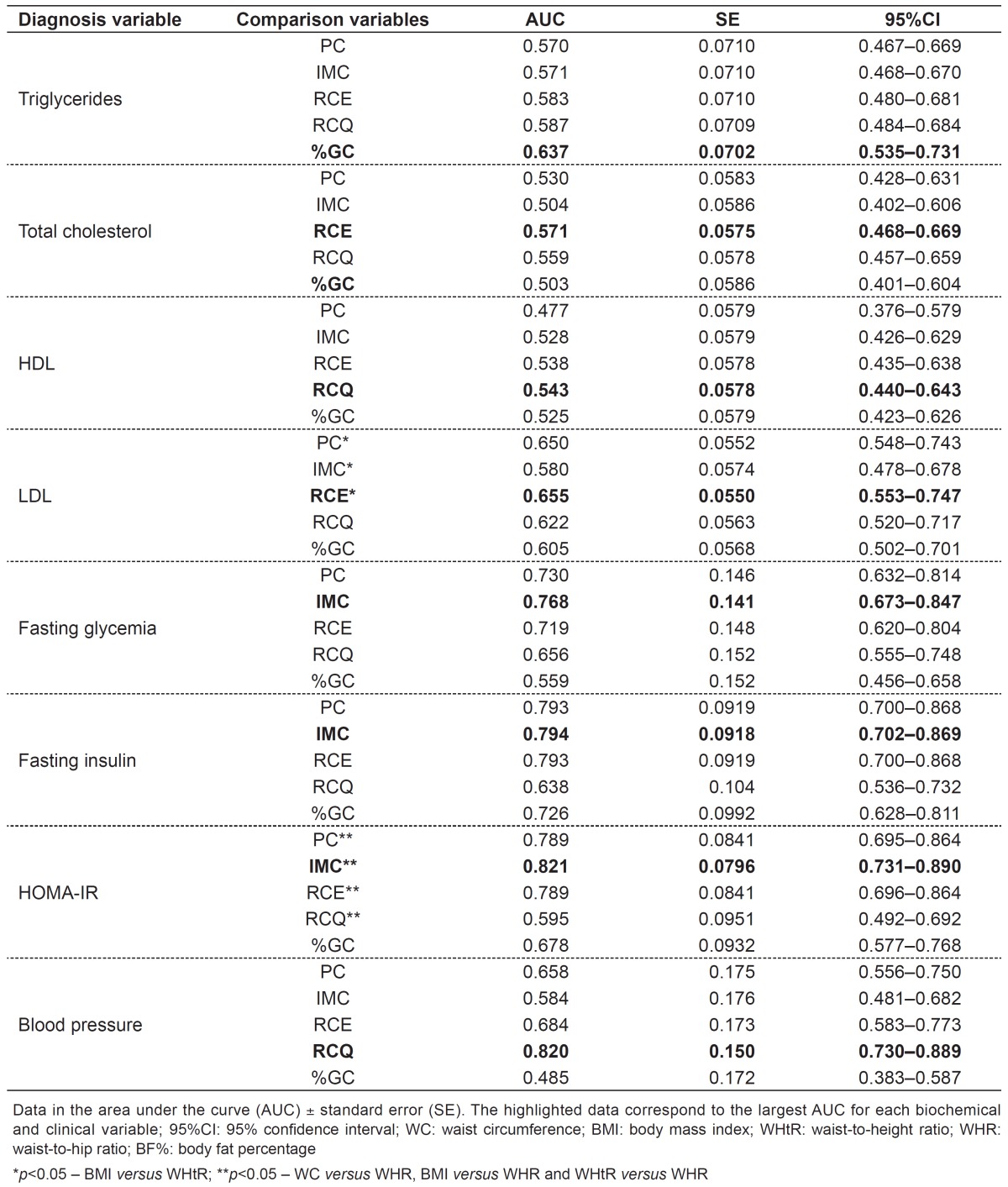
Results of the ROC curves comparing anthropometric and body composition variables as discriminators of alterations in lipid profile, glycemia, fasting insulin and glycemia, Homeostasis Model Assessment-Insulin Resistance, and blood pressure in female adolescents

BMI, WC, and WHtR were shown to be the best predictors for biochemical and clinical parameters and for the diagnosis of high HOMA-IR, glycemia, and fasting insulin levels. WHR was the best predictor for the diagnosis of arterial hypertension. Results of the Z-test showed that AUCs for WC, BMI, and WHtR were larger than the one for WHP for the diagnosis of HOMA-IR, and that the AUC for WHtR was larger than the one for BMI for the diagnosis of LDL alterations. The other AUCs did not present any difference.

**Figure 1:**
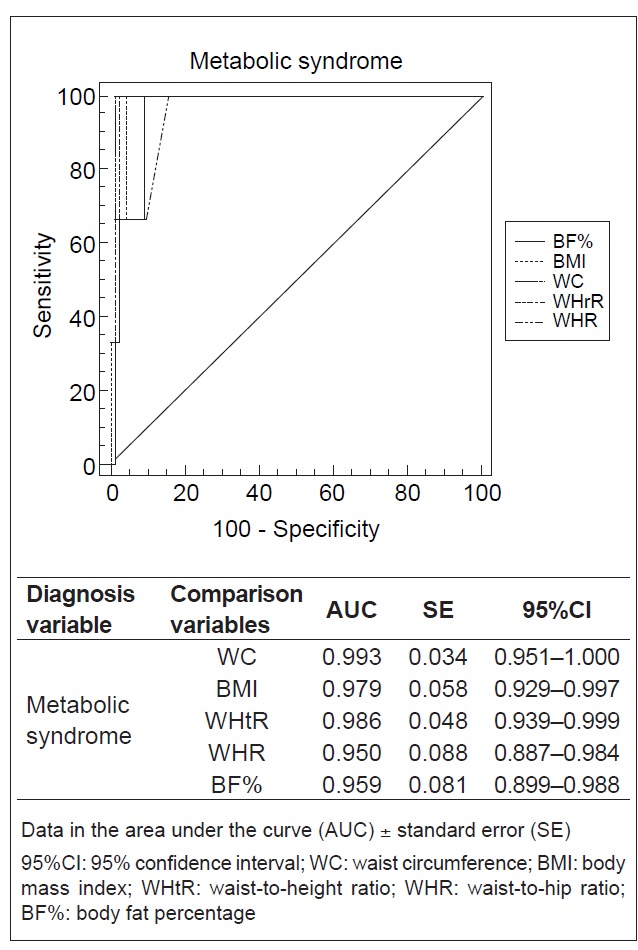
Results of the ROC curve comparing body composition and anthropometric variables as discriminators of metabolic syndrome in female adolescents

Regarding metabolic syndrome, the AUC for all anthropometric and body composition variables was next to 1.0, meaning they are effective for the diagnosis of this syndrome. The largest AUC was for WC. However, the Z-test showed no difference between AUC values (Figure 1). 

## Discussion

In this study, even though the BMI was suggestive of normal weight for most subjects, 96% of them showed one or more alterations in metabolic and body composition variables. These risk factors, when isolated, may induce atherosclerotic cardiovascular disease. The attempt to establish diagnostic criteria for metabolic syndrome is based on the idea that these componentes may act synergically or additively to increase the risk, which hasn't been demonstrated yet^(^ 19 ^)^. 

Anthropometric measurements are used as indicators of central body fat distribution in epidemiological studies aiming to identify adolescents at risk for cardiovascular diseases^(^ 4 ^-^ 6 ^)^. However, there is no consensus on the best anthropometric measurement for this, since a good indicator of the location of body fat should be associated with risk markers for cardiovascular diseases regardless of sex, age, and total adiposity.

The use of BMI for diagnosing obesity in adolescents is well documented in the literature, as it has a good clinical applicability. Besides, BMI shows an association with visceral fat and a better correlation with blood pressure and lipid levels when compared to other anthropometric parameters^(^ 20 ^)^. However, this indicator should not be used alone, since underweight or normal weight individuals do not always present adequate body fat percentage. The use of other instruments for this type of evaluation is therefore essential. In this study, out of all subjects who were not classified as overweight by the BMI (89%), 56.2% had excess body fat. These results are similar to another study with adolescent girls in which 56% of them were above the expected percentage of body fat, even though only 18% were overweight by BMI standards^(^ 21 ^)^. These findings stress the need for other anthropometric measurements to identify possible risk factors for a number of diseases. Rodríguez et al^(^ 22 ^)^ highlighted some anthropometric measurements, especially WC and electrical bioimpedance, as the most often used measurements.

BF% alone, assessed by horizontal bioimpedance, besides being well correlated with anthropometric measurements in this study, was correlated with insulin and insulin resistance, as well as with BMI. However, electrical bioimpedance has its limitations, such as the high cost and lack of knowledge on the equations used by the devices. This is why we chose to assess BF% using a validated equation proposed by Houtkooper et al^(^ 13 ^)^ for adolescents between ten and 19 years old which uses impedance data. Since the values of the equation were different from the ones provided by the device (*p*<0,001), we used the results given by the equation, as they had more correlations than the direct results obtained by biompedance. They were also able to diagnose a greater number of adolescents with excess body fat, which allows for the prevention of future risks.

Hence, due to the limitations of the above-mentioned methods for assessing central body fat, other anthropometric measurements are used in epidemiological studies, such as WC, WHR, and WHtR. These are cheaper and more practical^(^ 23 ^)^ when compared to more sensitive methods such as TC scans, MRIs, and DEXAs (Dual-energy X-ray absorptiometry), which would be economically unfeasible in this type of study.

WHtR is an increasingly used indicator and is shown to be a good marker for monitoring excess weight in adolescents^(^ 3 ^,^ 5 ^,^ 24 ^)^, since it considers growth in both height and waist^(^ 23 ^)^. Among all measurements studied in this article, WHtR was correlated with the greatest number of biochemical variables. Although these correlations were weak, they have great significance for clinical practice.

A study with 610 adolescents between 12 and 19 years old from public schools in Niterói, RJ, Brazil, evaluated the association between measurements of central body fat distribution and the components of metabolic syndrome. In boys, the positive association of WC and WHtR with triglycerides did not depend on BMI and BF%, respectively. WC was correlated with systolic blood pressure regardless of BF% in both sexes. The study concluded that WC was the measurement of central body fat that was best associated with the components of metabolic syndrome in adolescents^(^ 3 ^)^. WHR had the weaker association, having no significant effect on the investigated variables. This is corroborated by this study and by others from around the world^(^ 23 ^,^ 25 ^)^. Besides, WC, when used alone in adults, is accepted as an important tool for assessing the risk of some diseases, especially of atherosclerosis, and has shown a greater association with metabolic alterations than WHR. In adolescents, WHR does not seem to be appropriate as an anthropometric measurement to evaluate body fat distribution, since pelvic width changes rapidly during sexual maturation. Therefore, this index may be more related to physical changes during puberty than to actual body fat distribution^(^ 26 ^)^. However, there are no specific cutoff points for WC in Brazilian adolescents. This is why many studies focus on populations from other countries or conduct regional studies in order to establish cutoff points for this age group. Besides, this measurement varies due to physical growth, which means cutoff points, when existent, are different for each age group^(^ 27 ^)^. 

None of the anthropometric or body composition variables were able to diagnose alterations in total cholesterol and HDL. Beck et al^(^ 28 ^)^, in a study with adolescents between 14 and 19 years old from Três de Maio, RS, Brazil, found that BMI, WC, and WHtR in girls were not able to diagnose alterations in total cholesterol, but they were predictors of low HDL levels. 

Biochemical alterations may be present in individuals with adequate nutritional status, not only on those with excess weight. Gontijo et al^(^ 5 ^)^ evaluated 199 adolescents assisted by a healthcare program and found no difference in total cholesterol and LDL levels when compared to nutritional status, even though most of the subjects presented with these alterations. 

This study demonstrated that the upper quartile of BMI, WHtR, WC, and BF% showed greater values of fasting insulin and HOMA-IR when compared to the lower quartiles. This was also observed in the correlation test. Therefore, diagnosing insulin resistance is relevant to the assessment of the presence of metabolic syndrome. HOMA-IR has been widely used, representing one of the alternatives for the evaluation of these parameters. This is particularly true for studies involving a great number of participants, since it is a quick, practical, and cheap method^(^ 29 ^)^.

Regarding the ROC curve for variables, HOMA-IR, glycemia, and insulin resistance, the AUC values were greater for BMI, WC, and WHtR. Moreira et al^(^ 30 ^)^, in a study involving 109 children between seven and 11 years old from a public school in Taguatinga, DF, Brazil, found that, in the diagnosis of alteration in HOMA-IR, the largest AUC was for the BMI (AUC - 0.90/0.83-0.97), and the smallest one was for the WHR (AUC - 0.67/0.46-0.87). Besides, insulin resistance was associated with adolescence in a study with 196 children and adolescents between two and 18 years old from Campina Grande, PB, Brazil. It was also associated with altered triglyceride and HDL levels and with metabolic syndrome^(^ 31 ^)^. These results confirm that insulin resistance is already present in adolescence, and low-cost anthropometric measurements, such as BMI, WC, and WHtR, may predict this alteration. 

Unlike what has been said about HOMA-IR, insulin, and glycemia, the variable that showed the best diagnostic property for arterial hypertension was WHR, and AUC values close to 0.5 were found for WC, BMI, and BF%. This low predictive capacity of the WC and BMI for detecting hypertension was corroborated by a study with 1,201 adolescents from Londrina, PR, Brazil, in which obesity evaluated by BMI and WC had an AUC of 0.590 and 0.599, respectively^(^ 32 ^)^. 

All studied anthropometric and body composition variables were able to predict metabolic syndrome, with AUC values close to and above 0.90. This way, metabolic syndrome was the diagnosis variable that showed the largest AUC for all comparison variables. 

Therefore, it is important to adopt measurements that allow for the early diagnosis of these alterations^(^ 20 ^)^, and adolescence is an appropriate time to put these measurements into practice. Doing so will have a positive impact on the future, as this group is relevant and strategic for Public Health for their health promotion and disease prevention potential. It bears stressing that specific health care programs for adolescents are necessary.

Although some correlations are considered weak, results indicate that WC, BMI, BF%, and WHtR were able to predict insulin resistance, an alteration that should be monitored in adolescents. The higher the resistance level, the higher the prevalence of metabolic syndrome, which consequently increases the risk of early onset of cardiovascular diseases. These results are extremely important as they have great implications for Public Health. WHtR as a strong predictor of metabolic syndrome in female pubescent adolescents is new information for the scientific literature, which highlights the importance of new studies aiming to establish cutoff points for this index in adolescents.

However, some possible limitations of our study must be considered. One of them refers to data collection, which was performed over six years ago. There is, however, the need to study body composition variables as predictors of metabolic alterations, since the best predictor for this alteration in female pubescent adolescents is unknown. Considering that this study aimed mainly at evaluating whether body composition variables are able to predict metabolic alterations in adolescents, time of data collection does not affect the results, as this is a comparative study of body composition variables. The major limitation refers to the cutoff point used for HOMA-IR and WC. So far, there are no internationally-accepted cutoff points for these variables. 

We conclude that WC, BMI, and WHtR are good predictors of metabolic alterations in Brazilian female adolescents and should be used together in the nutritional assessment of this age group. These measurements are simple and low-cost, rendering them useful for both individual and collective assessments. The higher the number of indicators, the more reliable the nutritional diagnosis, which helps in the current and future prevention of metabolic alterations. 
