# Supplementary material for: Body composition and risk for metabolic alterations in female adolescents
Source: Rev Paul Pediatr. 2014 Jun;32(2):207–15. doi: 10.1590/0103-0582201432215313 (PMC4183020; doi:10.1590/0103-0582201432215313)
Supplement: Translation

# Translation: Composição corporal e risco de alterações metabólicas em adolescentes do sexo feminino

**Authors:** Eliane Rodrigues de Faria, Cristiana Araújo Gontijo, Sylvia do Carmo C. Franceschini, Maria do Carmo G. Peluzio, Silvia Eloiza Priore

**Affiliations:** 1 Universidade Federal do Espírito Santo (UFES), Alegre, ES, Brasil; 2 UFV, Viçosa, MG, Brasil

**Keywords:** composição corporal, adolescente, síndrome x metabólica, resistência à insulina.

## Abstract

**OBJETIVO:**

Estudar variáveis antropométricas e de composição corporal como preditores de risco de alterações metabólicas e de síndrome metabólica em adolescentes do sexo feminino.

**MÉTODOS:**

Coletaram-se dados bioquímicos, clínicos e de composição corporal de 100 adolescentes de 14 a 17 anos de escolas públicas de Viçosa, MG.

**RESULTADOS:**

Quanto ao estado nutricional, 83, 11 e 6% apresentaram eutrofia, sobrepeso/obesidade e baixo peso, respectivamente, e 61% apresentaram alto percentual de gordura corporal. O colesterol total foi o que apresentou maior porcentagem de inadequação (57%), seguido do HDL (*high-density lipoprotein - *50%), do LDL (*low-density lipoprotein -* 47%) e dos triglicerídeos (22%). Observou-se inadequação em 11, 9, 3 e 4% quanto à resistência à insulina, insulina de jejum, pressão arterial e glicemia, respectivamente. Encontraram-se maiores valores de insulina de jejum e HOMA-IR (índice de resistência à insulina, do inglês *Homeostasis Model Assessment-Insulin Resistance*) nos maiores quartis de índice de massa corpórea (IMC), perímetro da cintura, relação cintura/estatura e percentual de gordura corporal. Observou-se que o IMC, o perímetro da cintura e a relação cintura/estatura foram as variáveis que demonstraram predizer melhor o diagnóstico de níveis elevados de HOMA-IR, glicemia e insulina de jejum. A relação cintura/quadril se associou ao diagnóstico da hipertensão arterial. Todas as variáveis de composição corporal foram eficazes no diagnóstico da síndrome metabólica.

**CONCLUSÕES:**

As variáveis perímetro da cintura, IMC e relação cintura/estatura foram bons preditores de alterações metabólicas nas adolescentes e, por isso, devem ser usadas em conjunto para avaliar o estado nutricional nessa faixa etária.

## Introdução

A Organização Mundial da Saúde define a adolescência como o período dos dez aos 19 anos e envolve transformações físicas, psíquicas e sociais, as quais podem se manifestar de formas e em momentos diferentes para cada indivíduo^(^ ^1^ ^)^. A adolescência é um dos períodos críticos para o início ou a persistência da obesidade e para o desenvolvimento de suas complicações.

A obesidade na infância e na adolescência tem adquirido características epidêmicas mundialmente. Dados da Pesquisa de Orçamentos Familiares (POF), realizada em 2008-2009 em áreas metropolitanas do Brasil, encontraram 20,5% de excesso de peso em adolescentes de dez a 19 anos^(^ ^2^ ^)^. 

A avaliação de risco cardiovascular por meio de medidas antropométricas ainda é pouco investigada em adolescentes. Dentre as variáveis de composição corporal, destaca-se o percentual de gordura corporal, o perímetro da cintura e do quadril e as relações cintura/quadril e cintura/estatura, amplamente utilizados em estudos com adolescentes^(^ ^3^ ^-^ ^6^ ^)^.

Considerando-se que a instalação precoce dos fatores de risco cardiovascular aumenta a chance de doenças na vida adulta, a identificação de medidas antropométricas simples e não invasivas que se associem a esses fatores em adolescentes saudáveis é de grande utilidade para prevenir doenças crônicas não transmissíveis no futuro^(^ ^3^ ^)^. Sabe-se que o excesso de peso e/ou de gordura corporal pode aumentar os riscos de alterações metabólicas como dislipidemias, resistência à insulina, tolerância diminuída à glicose e hipertensão arterial, fatores que, quando presentes simultaneamente em um indivíduo, determinam o diagnóstico da síndrome metabólica^(^ ^4^ ^)^.

Desta forma, este estudo teve como objetivo estudar as variáveis de composição corporal como preditores de risco de alterações metabólicas e síndrome metabólica em adolescentes do sexo feminino. 

## Método

Estudo epidemiológico, de corte transversal. A população constituiu-se de 100 adolescentes do sexo feminino de 14 a 17 anos, selecionadas em escolas do ensino médio da rede pública do município de Viçosa, MG. Realizou-se a coleta de dados de setembro de 2006 a janeiro de 2007. Os critérios de inclusão foram: já ter apresentado menarca no mínimo há um ano, não ter doenças crônicas ou uso regular de medicamentos que alterassem a pressão arterial, a glicemia de jejum ou o metabolismo lipídico, não usar anticoncepcional há menos de dois meses e não fazer uso contínuo de diuréticos/laxantes, marcapasso ou prótese. 

A seleção da amostra baseou-se no total de adolescentes do sexo feminino na faixa etária de interesse, em 2006, matriculadas nas escolas da zona urbana de Viçosa^(^ ^7^ ^)^. Calculou-se a amostra por meio do programa Epi-Info, versão 6.04 para estudos transversais, considerando-se população total de 2.500 indivíduos, frequência esperada de síndrome metabólica de 8% e variabilidade de 2,5%, totalizando 90 indivíduos, com 95% de nível de confiança. A este acrescentaram-se 10% para recuperar possíveis perdas relacionadas à desistência das adolescentes. De todas as que atenderam aos critérios de inclusão (n=336), procedeu-se a uma seleção aleatória por sorteio, até que se obtivessem as 100 adolescentes. 

Obteve-se o peso em balança digital eletrônica, com capacidade máxima de 136kg e subdivisão em 100g, e a estatura por estadiômetro, com extensão de 2m, escala de 0,1cm, visor de plástico e esquadro acoplado a uma das extremidades. O peso e a estatura foram aferidos segundo as técnicas preconizadas pela Organização Mundial da Saúde (OMS)^(^ ^8^ ^)^. Avaliou-se o estado nutricional por meio do índice de massa corpórea (IMC), utilizando-se os pontos de corte e a referência antropométrica preconizados pela OMS^(^ ^9^ ^)^. Classificaram-se adolescentes com sobrepeso e obesidade como portadores de excesso de peso (≥ percentil 85)^(^ ^4^ ^,^ ^10^ ^)^. Os perímetros da cintura (PC) e do quadril (PQ) foram aferidos com fita métrica, com extensão de 2m, flexível e inelástica, dividida em centímetros e subdividida em milímetros, atentando-se para não comprimir as partes moles. Calculou-se a relação cintura/quadril (RCQ) e a relação cintura/estatura (RCE). O percentual de gordura corporal (%GC) foi estimado com aparelho de bioimpedância elétrica tetrapolar horizontal (Biodynamics^(r)^, modelo 310, versão 7.1), considerando-se os pontos de corte de Lohman^(^ ^11^ ^)^. Realizou-se a avaliação entre 7h e 8h30 da manhã, respeitando-se o protocolo de cuidados anteriores ao teste^(^ ^12^ ^)^. Utilizou-se fórmula validada proposta por Houtkooper *et al**^(13) ^*para adolescentes de dez a 19 anos, utilizando-se a resistência em ohms (Ω) obtida pela bioimpedância.

Aferiu-se a pressão arterial com monitor de pressão sanguínea de inflação automática, preconizado pela Sociedade Brasileira de Cardiologia, seguindo-se as orientações das V Diretrizes Brasileiras de Hipertensão Arterial^(^ ^14^ ^)^. Quanto aos pontos de corte para pressão sistólica e diastólica, consideraram-se os valores propostos segundo o percentil de estatura da adolescente. Coletou-se o sangue das voluntárias após jejum de 12 horas, para análise de glicemia, insulina plasmática e avaliação da concentração de lipídios séricos, como colesterol total, triglicerídeos, lipoproteína de alta densidade (HDL, do inglês *high-density lipoprotein*), lipoproteína de baixa densidade (LDL, do inglês *low-density lipoprotein*) e lipoproteína de muito baixa densidade (VLDL, do inglês *very low-density lipoprotein*). Para dislipidemias e insulina de jejum alterada (≥15µU/mL), os pontos de corte para adolescentes foram os preconizados pela I Diretriz de Prevenção da Aterosclerose na Infância e na Adolescência^(^ ^15^ ^)^. Para a glicemia de jejum alterada, utilizou-se a recomendação da *American Diabetes Association**^(^* ^16^ ^)^, que considera como critério a glicemia de jejum ≥100mg/dL. A resistência à insulina foi determinada pelo método *Homeostasis Model Assessment-Insulin Resistance* (HOMA-IR) - [insulina de jejum (µU/mL) x glicemia de jejum (mmol/L)/22,5]≥3,16^(^ ^17^ ^)^.

Para a classificação da síndrome metabólica, utilizou-se o critério proposto pela *International Diabetes Federation *(IDF)^(^ ^18^ ^)^, com os pontos de corte específicos da Sociedade Brasileira de Cardiologia para adolescentes^(^ ^15^ ^)^: perímetro da cintura ≥percentil 90 mais duas alterações: triglicerídeos ≥100mg/dL; HDL <45mg/dL; glicemia de jejum alterada ≥100mg/dL; pressão arterial ≥percentil 90 para estatura e sexo.

O projeto foi aprovado pelo Comitê de Ética em Pesquisa com Seres Humanos da Universidade Federal de Viçosa. A participação foi voluntária após esclarecimento verbal e por meio do termo de consentimento livre e esclarecido, obtendo-se a autorização pelos adolescentes e responsáveis.

Analisaram-se os dados com os *softwares* Epi-Info, versão 6.04, e *Sigma Statistic**^(r)^* *for Windows*. Utilizaram-se os seguintes testes: correlação de Pearson ou de Spearman, Análise de Variância (Anova) ou Teste de Kruskal-Wallis complementado pelo teste de comparação de Dunn ou teste de Tukey, dependendo da característica das variáveis. O nível de significância foi de 5% (*p*<0,05). Elaboraram-se curvas ROC (*receiver operating characteristic*) com o *software *Medcalc 9.03, para avaliar as variáveis de composição corporal capazes de predizer alterações bioquímicas, de pressão arterial e a síndrome metabólica. Calcularam-se as áreas abaixo das curvas (AUC) e seus respectivos intervalos de confiança de 95%. 

## Resultados

A média etária foi de 16,0±0,7 e a mediana, de 16 anos. A idade da menarca apresentou média de 12,3±1,1 anos, com mediana de 12,1 (mínimo de 10 e máximo de 15,1 anos). Quanto ao estado nutricional, 83, 11 e 6% apresentaram eutrofia, sobrepeso/obesidade e baixo peso, respectivamente, e 61% apresentaram alto percentual de gordura corporal. 

O colesterol total foi o marcador que apresentou maior percentual de inadequação (57%), seguido de HDL (50%), LDL (47%) e triglicerídeos (22%). A insulina de jejum estava alterada em 9% e a glicemia de jejum, em 4%. O HOMA-IR foi elevado em 11% e a pressão arterial inadequada, em 3%. A síndrome metabólica estava presente em 3% das adolescentes.

A comparação entre valores médios (desvio padrão - DP) e medianos de características metabólicas das adolescentes foi realizada segundo os quartis de IMC, PC, RCE, %GC e RCQ. A Tabela 1 apresenta apenas as variáveis bioquímicas que apresentaram diferença entre os quartis, observando-se maiores valores de insulina de jejum e HOMA-IR para os maiores quartis de IMC, PC, RCE e %GC e maiores valores de colesterol total/HDL e LDL/HDL para o maior quartil de %GC.

**Tabela 1:** Comparação entre os valores médios e medianos de características metabólicas das adolescentes estudadas conforme os quartis das variáveis de composição corporal

Encontrou-se correlação (*p*<0,05) entre RCE e HOMA-IR (r=0,337), insulina (r=0,327), colesterol total (r=0,256), LDL (r=0,264), colesterol total/HDL (r=0,265), LDL/HDL (r=0,281) e pressão sistólica (r=0,200); entre PC e HOMA-IR (r=0,332), insulina (r=0,312), pressão sistólica (r=0,238) e pressão diastólica (r=0,252); entre IMC e HOMA-IR (r=0,370), insulina (r=0,371) e pressão sistólica (r=0,226); entre %GC e HOMA-IR (r=0,286), insulina (r=0,303) e colesterol total (r=0,197); entre RCQ e colesterol total/HDL (r=0,238) e LDL/HDL (r=0,236).

Os resultados das curvas ROC (Tabela 2) demonstraram que as variáveis antropométricas e de composição corporal apresentaram menores AUC para o diagnóstico de níveis alterados de HDL e colesterol total, valores próximos de 0,5, demonstrando que essas variáveis possuem baixa eficácia para o diagnóstico da alteração dos níveis lipídicos. 

**Tabela 2:** Resultados das curvas ROC comparando variáveis antropométricas e de composição corporal enquanto discriminadores de alterações no perfil lipídico, glicemia e insulina de jejum, *Homeostasis Model Assessment–Insulin* Resistance e pressão arterial em adolescentes do sexo feminino

As variáveis que demonstraram ser melhores preditores dos parâmetros bioquímicos e clínicos foram IMC, PC e RCE, para o diagnóstico de níveis elevados de HOMA-IR, e glicemia, insulina de jejum e RCQ, para o diagnóstico da hipertensão arterial. Os resultados do teste Z demonstraram que as AUC para as variáveis PC, IMC e RCE foram maiores do que a RCQ no diagnóstico do HOMA-IR e que a AUC para a RCE foi maior que a do IMC no diagnóstico de alteração do LDL. As demais não apresentaram diferença.

Quanto à síndrome metabólica, todas as variáveis antropométricas e de composição corporal apresentaram AUC próximo de 1,0, demonstrando serem eficazes no diagnóstico da síndrome. Entretanto, o maior valor AUC foi para o PC, porém, por meio do teste Z, não houve diferença entre as AUC (Figura 1). 

**Figura 1:** Resultados da curva ROC comparando variáveis antropométricas e de composição corporal enquanto discriminadores da síndrome metabólica em adolescentes do sexo feminino

## Discussão

Neste estudo, destaca-se que, mesmo diante da predominância da eutrofia pelo IMC, 96% apresentaram uma ou mais alterações relacionadas às variáveis de composição corporal e metabólicas. Esses fatores de risco, isoladamente, têm a capacidade de induzir à doença cardiovascular aterosclerótica. As tentativas de estabelecer critérios diagnósticos para a síndrome metabólica são baseadas no princípio de que tais componentes podem agir de maneira sinérgica ou aditiva ampliando o risco, o que ainda não se demonstrou^(^ ^19^ ^)^. 

As medidas antropométricas são utilizadas como indicadoras de localização de gordura central em estudos epidemiológicos que visam identificar adolescentes com risco para doenças cardiovasculares^(^ ^4^ ^-^ ^6^ ^)^. No entanto, ainda não há consenso sobre a melhor medida para esse fim, uma vez que um bom indicador de localização de gordura corporal deveria associar-se, independentemente de sexo, idade e adiposidade total, com os marcadores de risco para doenças cardiovasculares^(^ ^3^ ^)^.

O uso do IMC no diagnóstico da obesidade em adolescentes está bem estabelecido na literatura, pois tem boa aplicabilidade clínica. Além disso, o IMC demonstra associação com gordura visceral e melhor correlação com pressão arterial e níveis lipídicos do que outros parâmetros antropométricos^(^ ^20^ ^)^. Porém, ressalta-se que esse indicador não deve ser usado isoladamente, uma vez que nem sempre os indivíduos considerados como de baixo peso e eutróficos possuem percentual de gordura corporal adequado, o que torna indispensável a aplicação de outros instrumentos para tal avaliação. Neste estudo, dos 89% sem excesso de peso pelo IMC, 56,2% apresentaram excesso de gordura corporal, de modo similar a uma pesquisa também realizada com meninas, em que, embora apenas 18% apresentassem excesso de peso pelo IMC, 56% contavam com percentual de gordura acima do esperado^(^ ^21^ ^)^. Tais achados reforçam a necessidade de se avaliarem outras medidas antropométricas para identificar possíveis fatores de risco à saúde. Rodríguez *et al**^(22) ^*destacam as medidas antropométricas, principalmente o PC e a bioimpedância elétrica, como as mais frequentemente utilizadas.

O %GC, isoladamente, aferido pela bioimpedância horizontal, além de estar bem correlacionado com as variáveis antropométricas neste estudo, também se correlacionou à insulina e à sua resistência, assim como o IMC. Porém, a bioimpedância elétrica tem suas limitações, como o alto custo e a falta de conhecimento das fórmulas utilizadas nos equipamentos. Por isso, neste estudo, optou-se por utilizar o %GC avaliado por meio de fórmula validada para adolescentes de dez a 19 anos, proposta por Houtkooper *et al**^(^* ^13^ ^)^, que utiliza, em sua equação, os dados de impedância. Como os valores dessa fórmula foram diferentes daqueles obtidos pelo equipamento (*p*<0,001), utilizaram-se os resultados apresentados pela fórmula, uma vez que se obtiveram maiores correlações do que os resultados diretos dados pela bioimpedância, além de serem capazes de diagnosticar maior número de adolescentes com excesso de gordura corporal, possibilitando a prevenção de riscos futuros.

Dessa forma, pelas limitações dos métodos anteriormente citados para avaliar a gordura corporal na região central, utilizam-se outras medidas antropométricas em estudos epidemiológicos, como PC, RCQ, RCE, pela praticidade e pelo baixo custo^(^ ^23^ ^)^, já que métodos mais sensíveis, como a tomografia computadorizada, a ressonância magnética e o DEXA (*Dual-energy X-ray absorptiometry*) são inviáveis do ponto de vista econômico nesse tipo de estudo.

A RCE é um indicador de uso crescente, mostrando-se bom marcador para monitorar excesso de peso em adolescentes^(^ ^3^ ^,^ ^5^ ^,^ ^24^ ^)^ porque considera o crescimento tanto da cintura quanto da estatura^(^ ^23^ ^)^. Neste estudo, a RCE foi a medida que teve correlação com maior número de variáveis bioquímicas e, embora as correlações tenham sido fracas, são de grande significado na prática clínica.

Estudo com 610 adolescentes de 12 a 19 anos de escolas públicas de Niterói, RJ, avaliou a associação entre medidas de localização de gordura central com os componentes da síndrome metabólica. Nos meninos, a associação positiva entre PC e RCE com triglicerídeos foi independente do IMC e do %GC, respectivamente. O PC correlacionou-se com a pressão arterial sistólica independentemente do %GC, em ambos os sexos. Concluiu-se que o PC foi a medida de gordura central que apresentou a melhor associação com os componentes da síndrome metabólica nos adolescentes^(^ ^3^ ^)^. A RCQ foi a medida que mostrou a pior associação, não tendo efeito significativo para nenhuma das variáveis investigadas, similar ao encontrado neste estudo e também em outros países do mundo^(^ ^23^ ^,^ ^25^ ^)^. Além disso, o PC isolado em adultos é aceito como ferramenta importante para avaliar o risco de doenças, especialmente da aterosclerose, e tem demonstrado melhor associação com as alterações metabólicas do que a RCQ. Em adolescentes, parece que a RCQ não é apropriada como medida antropométrica para avaliar a distribuição da gordura corporal, pois a largura pélvica sofre alterações rápidas durante a maturação sexual, podendo esse índice estar mais relacionado às modificações corporais da puberdade do que à distribuição da gordura corporal propriamente dita^(^ ^26^ ^)^. Porém, não há pontos de corte específicos de PC em adolescentes brasileiros. Por isso, muitos estudos utilizam populações de outros países ou realizam estudos regionais a fim de estabelecer pontos de corte para essa faixa etária. Além disso, essa medida apresenta variação em razão do crescimento físico, fazendo com que os pontos de corte, quando existem, sejam diferentes para cada faixa etária^(^ ^27^ ^)^. 

Nenhuma das variáveis antropométricas e de composição corporal foi capaz de diagnosticar alterações no colesterol total e na HDL. Beck *et al**^(^* ^28^ ^)^, em estudo com adolescentes de 14 a 19 anos do município de Três de Maio, RS, verificaram que, nas meninas, as variáveis IMC, PC e RCE não foram capazes de diagnosticar alterações no colesterol total, porém predisseram baixos níveis de HDL. 

As alterações bioquímicas podem estar presentes em indivíduos com estado nutricional adequado, não somente naqueles com excessos de peso. Gontijo *et al**^(5) ^*avaliaram 199 adolescentes atendidos em um programa de saúde e não houve diferença nos valores de colesterol total e LDL quando comparados quanto ao estado nutricional, apesar de grande parte dos indivíduos ser portador dessas alterações. 

O presente estudo demonstrou que o último quartil do IMC, da RCE, do PC e do %GC apresentava valores maiores de insulina de jejum e HOMA-IR comparado aos primeiros quartis, o que também foi observado pelo teste de correlação. Portanto, o diagnóstico da resistência à insulina é relevante na avaliação da presença de síndrome metabólica. O HOMA-IR tem sido amplamente utilizado, representando uma das alternativas para avaliar esses parâmetros, principalmente em estudos envolvendo grande número de participantes, por ser um método rápido, de fácil aplicação e de menor custo^(^ ^29^ ^)^.

Quanto à curva ROC para variáveis, diagnóstico HOMA-IR, glicemia e insulina de jejum, os valores de AUC foram maiores para IMC, PC e RCE. Moreira *et al**^(^* ^30^ ^)^, em estudo com 109 indivíduos de sete a 11 anos de escola pública de Taguatinga, DF, observaram que, para o diagnóstico de alteração no HOMA-IR, o maior valor de AUC foi para o IMC (AUC - 0,90/0,83-0,97) e o menor valor, para a RCQ (AUC - 0,67/0,46-0,87). Além disso, a resistência à insulina associou-se à adolescência em estudo com 196 crianças e adolescentes de dois a 18 anos de Campina Grande, PB, tendo se associado também a níveis alterados de triglicerídeos, HDL e síndrome metabólica^(^ ^31^ ^)^. Esses resultados confirmam que, na adolescência, a resistência à insulina já está presente e medidas antropométricas de baixo custo, como IMC, PC e RCE, podem predizer tal alteração.

Ao contrário do apresentado para HOMA-IR, insulina e glicemia, a variável que demonstrou melhor propriedade diagnóstica para a hipertensão arterial foi a RCQ e valores de AUC próximos de 0,5 foram encontrados para PC, IMC e %GC. Essa baixa capacidade preditiva de PC e IMC para detectar hipertensão também foi encontrada em estudo com 1.021 adolescentes de Londrina, PR, sendo que a obesidade avaliada pelo IMC e pelo PC apresentou AUC de 0,590 e 0,599, respectivamente^(^ ^32^ ^)^. 

Todas as variáveis antropométricas e de composição corporal estudadas foram capazes de predizer a síndrome metabólica, mostrando valores de AUC próximos e superiores a 0,90. Assim, a síndrome metabólica foi a variável diagnóstica que apresentou maiores AUC para todas as comparações.

Desse modo, é importante adotar medidas que permitam o diagnóstico precoce dessas alterações^(^ ^20^ ^)^. Destaca-se a adolescência como momento oportuno para colocar em prática essas medidas, a fim de que tenham impacto positivo no futuro, uma vez que esse grupo é relevante e estratégico em termos de Saúde Pública, para promoção de saúde e prevenção de doenças. Reforça-se, portanto, a necessidade de programas específicos de atenção à saúde dos adolescentes.

Apesar de algumas correlações serem consideradas fracas, os resultados indicam que as medidas antropométricas PC, IMC, %GC e RCE foram capazes de predizer a resistência à insulina, que deve ser monitorada em adolescentes, pois quanto maior o nível de resistência à insulina, maior a prevalência de síndrome metabólica e, consequentemente, maior o risco de desenvolvimento prematuro de doenças cardiovasculares. Tais resultados são extremamente importantes, com grandes implicações na Saúde Pública. A RCE como um forte preditor de síndrome metabólica em adolescentes do sexo feminino, púberes, é uma informação nova em relação ao que já vem sendo estudado pela literatura científica, reforçando a importância de pesquisas para estabelecer pontos de corte desse índice em adolescentes.

Entretanto, devem-se considerar algumas limitações do presente estudo. Uma delas poderia se referir à data da coleta de dados, que já ultrapassou seis anos. Há, contudo, a necessidade de estudar as variáveis de composição corporal na predição de alterações metabólicas, uma vez que não se sabe qual é a melhor preditora dessas alterações em adolescentes púberes do sexo feminino. Como o objetivo principal do estudo foi avaliar se as variáveis de composição corporal são capazes de predizer alterações metabólicas nos adolescentes, a época da coleta de dados não compromete os resultados apresentados, já que se trata de um estudo de comparação entre as variáveis de composição corporal. A limitação mais importante do estudo refere-se, seguramente, ao ponto de corte utilizado para o indicador HOMA-IR e para o PC. Até a presente data, não existem pontos de corte internacionalmente aceitos para essas variáveis. 

Portanto, conclui-se que o PC, o IMC e a RCE são bons preditores de alterações metabólicas em adolescentes brasileiras do sexo feminino e, por isso, devem ser usados em conjunto na avaliação nutricional nessa faixa etária. Essas são medidas simples e de baixo custo, o que facilita sua utilização em nível individual e populacional. Quanto maior o número de indicadores usados, mais confiável será o diagnóstico nutricional, o que auxilia, portanto, na prevenção dessas alterações metabólicas na vida atual e futura.
